# Integrated analysis of single-cell RNA-seq and bulk RNA-seq unravels the heterogeneity of cancer-associated fibroblasts in TNBC

**DOI:** 10.18632/aging.205205

**Published:** 2023-11-13

**Authors:** Xiaoqing Wu, Wenping Lu, Weixuan Zhang, Dongni Zhang, Heting Mei, Mengfan Zhang, Yongjia Cui, Zhili Zhuo

**Affiliations:** 1 Department of Oncology, Guang'anmen Hospital, China Academy of Chinese Medical Sciences, Beijing 100053, People's Republic of China

**Keywords:** triple-negative breast cancer, heterogeneity, cancer-associated fibroblasts, bioinformatic

## Abstract

Background: The treatment of triple-negative breast cancer (TNBC) is one of the main focuses and key difficulties because of its heterogeneity, and the source of this heterogeneity is unclear.

Methods: Single-cell RNA (scRNA) and transcriptomics data of TNBC and normal breast samples were retrieved from Gene Expression Omnibus (GEO) database and TCGA-BRCA database. These cells were clustered using the t-SNE and UMAP method, and the marker genes for each cluster were found. We annotated the clusters using the published literature, CellMarker database and “SingleR” R package.

Results: A total of 1535 cells and 21785 genes from 6 TNBC patients and 2068 cells and 15868 genes from 3 normal breast tissues were used for downstream analyses. The scRNA data were divided into 14 clusters labeled into 8 cell types, including epithelial cells, immunocytes, CAFs/fibroblasts and etc. In the TNBC samples, CAFs were divided into three clusters and labelled as prCAFs, myCAFs and emCAFs, and the marker genes were DCN, FAP and RGS5, respectively. The prCAF subgroup is functionally characterized by promoting proliferation and multi drug resistance; myCAF subgroup is involved in constituting the extracellular matrix and collagen production, matrix composition and collagen production, and the emCAF functionally characterized by energy metabolism.

Conclusions: TNBC has inter- and intra-tumor heterogeneity, and CAF is one of the sources of this heterogeneity. CD74, SASH3, CD2, TAGAP and CCR7 served as significant marker genes with prognostic and therapeutic value.

## INTRODUCTION

Female breast cancer has surpassed lung cancer as the most prevalent cancer, with an estimated 2.3 million new cases (11.7%), and triple-negative breast cancer (TNBC) accounts for 11%-20% of all breast cancers [1]. It is characterized by the lack of estrogen receptor, progesterone receptor and human epidermal growth factor receptor 2 expression, as assessed by immunohistochemistry [2]. Its biological aggressiveness and propensity to metastasize are higher than those of any other pathological type of breast cancer [3]. Patients with TNBC have a poor prognosis as well [4]. Therapeutic approaches to TNBC are limited by the lack of therapeutic targets. Conventional chemotherapy, consisting mainly of anthracyclines and paclitaxel, has been the mainstay of treatment [5]. However, the heterogeneity of TNBC contributes to reducing the effectiveness of chemotherapy [6]. Although the majority of TNBC patients receive conventional chemotherapy, some patients will still experience recurrent metastases. This suggests that eliminating most cancer cells in tumor tissue does not inhibit progression [7]. A study showed that multiclonal seeding from individual clones in TNBC tissue leads to multisite metastasis [8]. Oncogene mutations, amplifications, loss-of-function mutations in tumor suppressors, and large-scale chromosomal alterations are all recognized mechanisms that have previously been shown to drive cancer evolution and generate subpopulations of cancer cells for metastatic spread and growth [9, 10]. However, recent advances suggest that epigenetics and transcriptional reprogramming are now considered key factors driving tumor heterogeneity and evolution [11]. Single-cell RNA sequencing (scRNA-seq) uses optimized next-generation sequencing technology to define the global gene expression profile of individual cells, which helps isolate heterogeneity previously hidden in cell populations [12]. In a patient-derived breast cancer xenograft model, single-cell RNA sequencing revealed that both primary tumors and micro-metastases show transcriptional heterogeneity that is highly predictive of poor patient survival [13]. These results may be evidence of tumor progression guided by tumor heterogeneity. Therefore, identifying the diversity of TNBC cells and subsets of associated cells at the single-cell level will contribute to the precise treatment of TNBC.

The tumor microenvironment, which includes the surrounding cells and molecules that interact with cancer cells, plays a critical role in the development of tumors, including the occurrence, progression, and immune suppression of the tumors [14, 15], cancer-associated fibroblasts are an important part of that. CAFs as the prominent stromal cell type in solid tumors, can enhance tumor phenotypes, especially cancer cell proliferation and invasion, neoangiogenesis, inflammation and extracellular matrix (ECM) remodeling [16, 17]. Abundant stromal myofibroblasts are associated with aggressive adenocarcinoma in human breast tumors and predict disease recurrence in humans [18]. In addition, the infiltration of CAFs contributes to angiogenesis, drug resistance and reduces antitumor immunity [3, 19, 20]. Most studies have focused on the relationship between CAFs and the tumor microenvironment and the immune cells within it, while its heterogeneity in human cancers is far from complete. In human TNBC samples with single-cell sequencing, CAFs clustered into two states: the first with features of myofibroblasts, and the second with high expression of growth factors and immunomodulatory molecules [21]. A study classified CAFs in human breast cancer into four subpopulations with different properties and activation levels. Two subpopulations of myofibroblasts with immunosuppressive effects (CAF-S1 and CAF-S4) accumulate differentially in TNBC [22]. Three CAF subpopulations were identified in 4T1 tumors transplanted from BALB/c mice, including myofibroblastic CAFs, enriched in α smooth muscle actin and several other contractile proteins; inflammatory CAFs with elevated expression of inflammatory cytokines; and CAF subpopulations expressing major histocompatibility complex class II proteins [23]. It has been previously shown that CAFs express high levels of alpha-smooth muscle actin (α-SMA/Acta2), fibroblast activation protein (Fap), fibroblast-specific protein 1 (S100a4), CD74, etc. [24, 25]. However, these markers are not exhaustive or can characterize certain subgroups. Therefore, the use of single-cell analysis is necessary to identify intra- and inter-tumor heterogeneity in TNBC tumors. Based on the above, we proposed the hypothesis that CAFs in TNBC are heterogeneous and that this heterogeneity may contribute to differences in patient prognosis.

In the present study, to explore the heterogeneity of CAFs in TNBC and the prognostic value of CAFs, we identified the biomarkers and functions of three subpopulations of CAFs and molecular fractionation of TNBC through scRNA-seq and bulk RNA-seq, described the tumor heterogeneity in TNBC and developed a prognostic model based on CAF-related genes.

## MATERIALS AND METHODS

### Data extraction

scRNA-seq was retrieved from GSE75688, GSE118389 and GSE226391 in the Gene Expression Omnibus (GEO) database (https://www.ncbi.nlm.nih.gov/geo/). A total of 21785 genes and 1534 cells were obtained in 6 TNBC patients and 21921 genes and 4840 cells in 3 normal breast tissues. Transcriptomics data from GSE19615 and GSE21653 in the GEO database and 107 TNBC patients were screened by clinical information. Transcriptomics data of 142 TNBC patients were screened by corresponding clinical information from TCGA-BRCA in the TCGA database (https://portal.gdc.cancer.gov/). RNA-seq data were normalized, and batch effects were removed by the R packages “limma” and “sva”.

### scRNA-seq quality control and analyses

The extracted data need to be quality controlled. Data quality control and analysis were applied using the “seurat” R package. First, the percentage of mitochondrial genes was tested, and a percentage less than 5% was considered satisfactory for cell quality control [26]. Second, the sequencing depth and counts of each cell were detected, and low-quality single-cell data were removed. Third, the first 1500 highly variable genes were extracted for subsequent analysis. After that, PCA was performed for linear dimensionality reduction. These cells were clustered using the louvain clustering method, and the marker genes for each cluster were found. In the analysis for the TNBC samples, the PC value is defined as 14 according to the ElbowPlot function, as shown in Supplementary Figure 1. The resolution is defined as 0.5 according to the “clustree” package, as shown in Supplementary Figure 2. In the analysis for the normal tissue, the PC value is defined as 9, the resolution is defined as 0.5, as shown in Supplementary Figures 3, 4. K-nearest neighbor value defaults to 20 according to the number of cells. We annotated the clusters using the published literature, the CellMarker database (http://xteam.xbio.top/CellMarker/index.jsp) and referring to the annotation results of the “SingleR” R package. The marker genes for each cluster were considered to be the most significantly different genes in PCs. Tumor cells and normal cells were identified by the R package “copykat”. The distribution of cell types for each sample was counted and visualized. Finally, cell trajectory analysis was performed using the “monocle” R package to derive the trajectory for each type of cell in terms of state, clustering and pseudotime distribution to find the differential genes on each branch. The results of each of the above steps were visualized.

### Biological functional and signaling pathway analyses

To identify the function of each CAF subgroup, Gene Ontology (GO) and Kyoto Encyclopedia of Genes and Genomes (KEGG) analyses were performed using the genes and expressions of each subgroup. GO and KEGG enrichment analyses using cluster marker genes were performed with R with the aid of the packages clusterProfiler, enrichplot, and ggplot2. Terms with FDR values of < 0.05 were considered significantly enriched. GO analyses included GO biological process, GO molecular function and GO cellular component. The top 10 terms of all of the above analyses were visualized by barplot.

### Identification of molecular subtypes

We intended to cluster the sample with the inner feature in tumor samples and extracted biological correlation coefficients, and the R package “ConsensusClusterPlus” was used to take the above action. We selected an optimum k value of 2 to 9 in consideration of stability and clustering performance. A suitable k value was chosen based on consensus CDF and delta area. Kaplan-Meier analysis was conducted between clusters via the “survival” R package. We also examined the differences in clinical characteristics in clusters and visualized them.

### Definition and comparison of the tumor immune infiltration microenvironment (TIME)

We used ESTIMATE to assess the tumor microenvironment by the R package “estimate” and obtained ESTIMATE scores, immune scores, stromal scores and tumor purity. By using the CIBERSORT algorithm, we calculated the absolute abundance of 22 types of immune cells. By contrasting the scores between clusters, it was possible to identify the infiltrating cells. The “ggpubr” package was used to visualize the results above. Differences in common immune checkpoints between clusters were analyzed by the R package “limma”. Kaplan-Meier analysis was conducted between clusters via the “survival” R package.

### Construction and validation of CAF-related gene prognostic features

CAF signature genes were found in the pseudotime analysis, which were used to build prognostic prediction model. To screen out CAF-related genes that may be related to prognosis, we performed Cox regression with the R packages “survival” and “survminer” to obtain candidate genes. The Lasso algorithm was executed to construct a prognostic model by the “glmnet” R package. We determined the risk score using the formula:


$$Risk\;score=\sum_{i=1}^{n}\left(coefi*Expi\right)$$

The mathematical meaning of the representation of this formula is to calculate the expression of each candidate gene multiplied by the regression coefficient of the multivariate Cox and then to sum all the values. We divided all cases into a high-risk group and a low-risk group based on the median risk score. The training set was composed of GEO data, and the testing set was composed of TCGA data. Receiver operating characteristic (ROC) curve analysis was used to confirm that the prognostic model was stable. To establish the risk score as a significant prognostic factor, univariate and multivariate Cox regression was conducted.

### Establishment and verification of the nomogram

The R program “rms” was used to finish the establishment, and “regplot” was used to complete the visualization. The model was built taking into account nomogram risk scores and clinical features. We offer a measurable tool to forecast overall survival at 1, 3, and 5 years. Calibration curves were created to evaluate the effectiveness of the nomogram.

### Extraction of hub genes

Hub genes, which are high-degree genes, have high connectivity in the protein-protein interaction network. The PPI network was constructed by the STRING database (https://cn.string-db.org/) based on CAF-related genes, followed by reconstruction with Cytoscape version 3.6.1. Nodes with confidence of interactive relationship larger than 0.95 were used for building the network. The number of adjacent nodes of each gene was counted by R.

### Statistical analysis

All the data were processed and analyzed by using R software (version: 4.1.2). In comparisons of two classes of data, we used the Mann-Whitney U test (for nonnormally distributed data) or Student’s t test (for normally distributed data). Spearman’s correlation test (for nonnormally distributed data) or Pearson’s correlation test (for normally distributed data) was applied to evaluate the correlation between two groups of data. We used Fisher’s exact or chi-square tests to assess the association between two categorical variables. We utilized the FDR, evaluated by the Benjamini-Hochberg method, to adjust for multiple tests.

### Availability of data and materials

All data generated or analysed during this study are included in this published article.

## RESULTS

### Normalization and dimensionality reduction of scRNA-seq data

**Figure 1:**
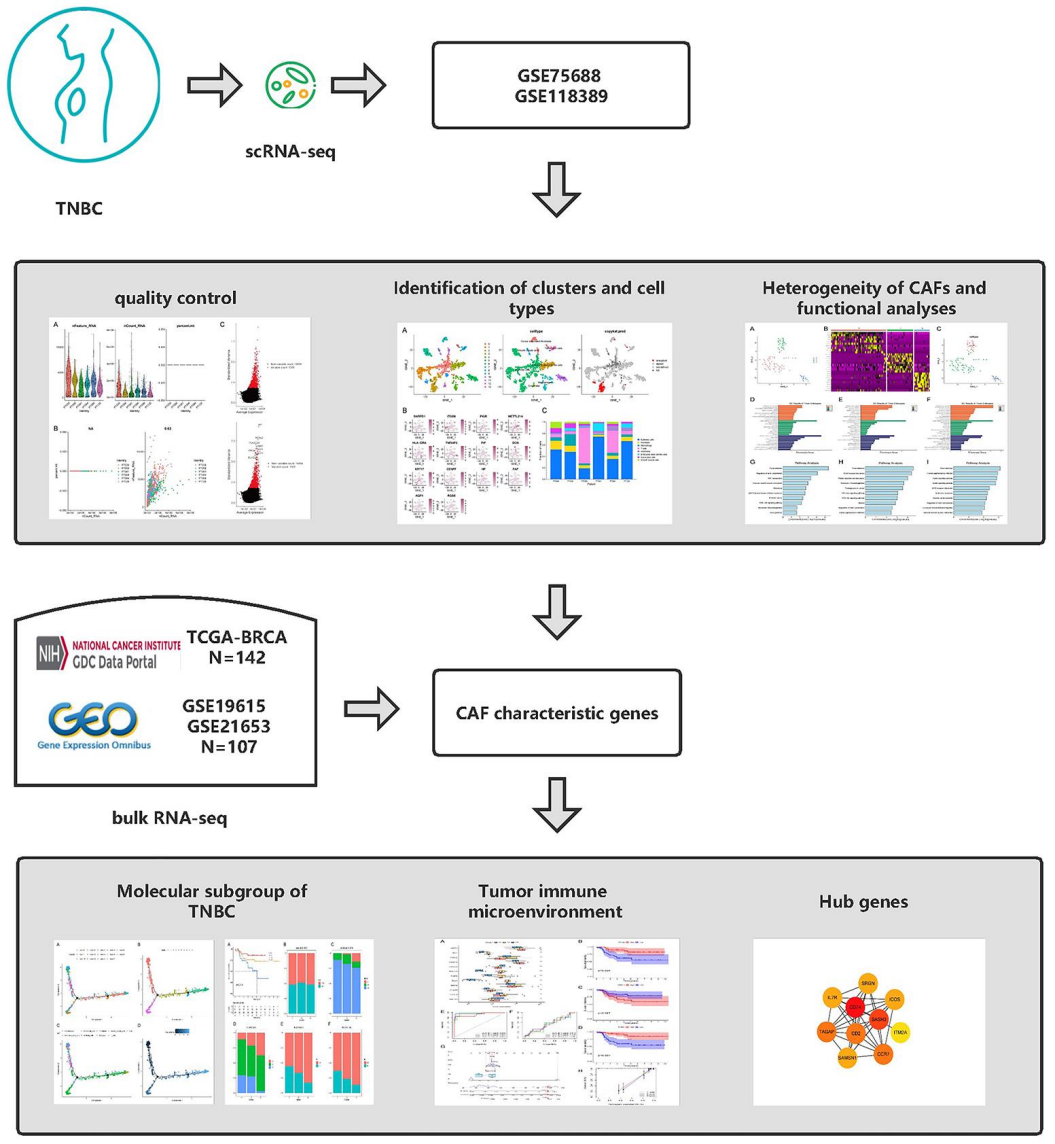
Graphical abstract of the analysis process.

**Figure 2:**
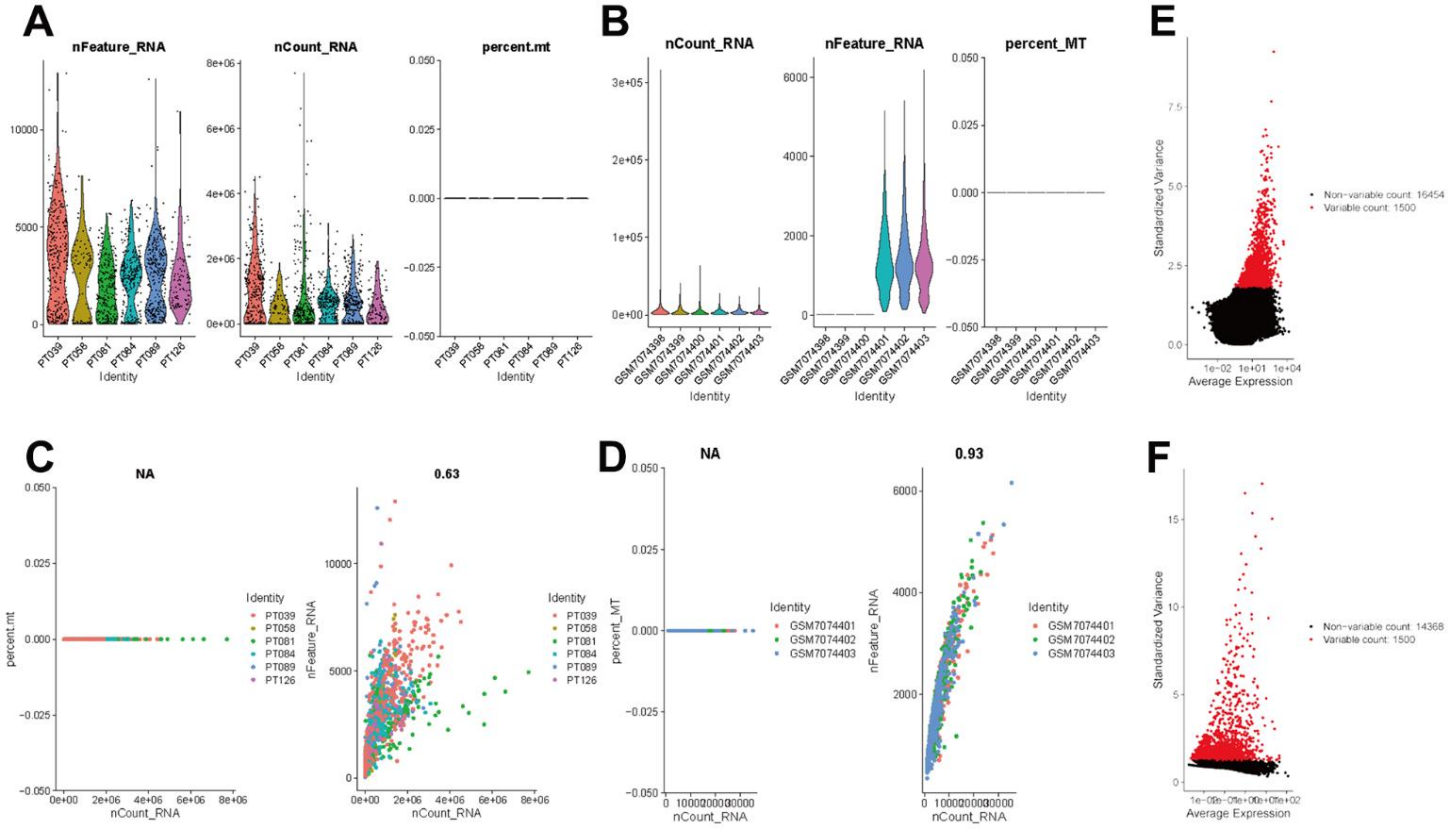
**Normalization and quality control of scRNA-seq.** (**A**) Single-cell sequencing depth, counts and fraction of reads mapped to mitochondrial genes in TNBC samples. (**B**) Single-cell sequencing depth, counts and fraction of reads mapped to mitochondrial genes in breast tissues. (**C**) Correlation of gene count and percent of mitochondrial genes and features in TNBC. (**D**) Correlation of gene count and percent of mitochondrial genes and features in breast tissue. (**E**) The first 1500 genes were screened as variant genes in TNBC samples. (**F**) The first 1500 genes were screened as variant genes in normal breast.

The flow of analyses in the presented study is shown in Figure 1. In the GSE226391, three samples, GSM7074398, GSM7074399 and GSM7074340, were of too low quality and were therefore excluded. Ultimately, a total of 1535 cells and 21785 genes from 6 TNBC patients and 2068 cells and 15868 genes from 3 normal breast tissues were used for downstream analyses. Visualization of sequencing depth, gene quantity, and mitochondrial gene content showed that scRNA data were available, as shown in Figure 2A, 2B. The percent of mitochondrial genes was independent of the counts of genes, and the sequencing depth was positively correlated with the counts of genes with a coefficient of 0.63 and 0.93 in TNBC and normal breast individually (Figure 2C, 2D). The relationship between normalized variance and average expression of all genes for each cell was visualized as shown in Figure 2E, 2F, and the 1500 genes with the largest normalized variables were selected for subsequent analysis.

### Identification of clusters and cell types

**Figure 3:**
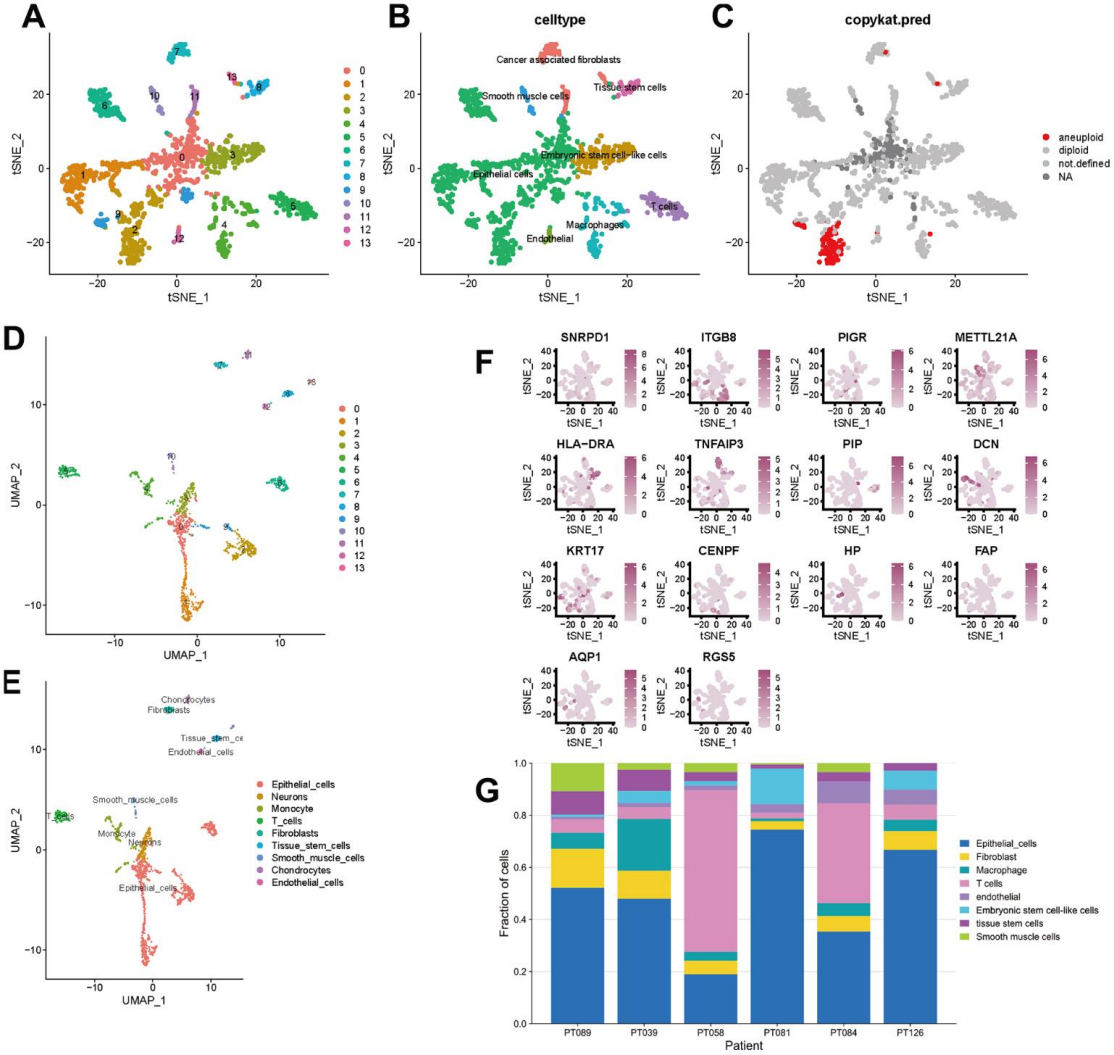
**t-SNE and UMAP clustering of TNBC samples.** (**A**–**C**) Clusters, cell type annotations and aneuploid cells at the single-cell level in TNBC samples by t-SNE method. (**D**, **E**) Clusters and cell type annotations at the single-cell level in TNBC samples by UMAP method. (**F**) Scatter plot of marker gene expression in each cluster. (**G**) Inter-tumor heterogeneity of triple-negative breast cancer at the single-cell level.

**Figure 4:**
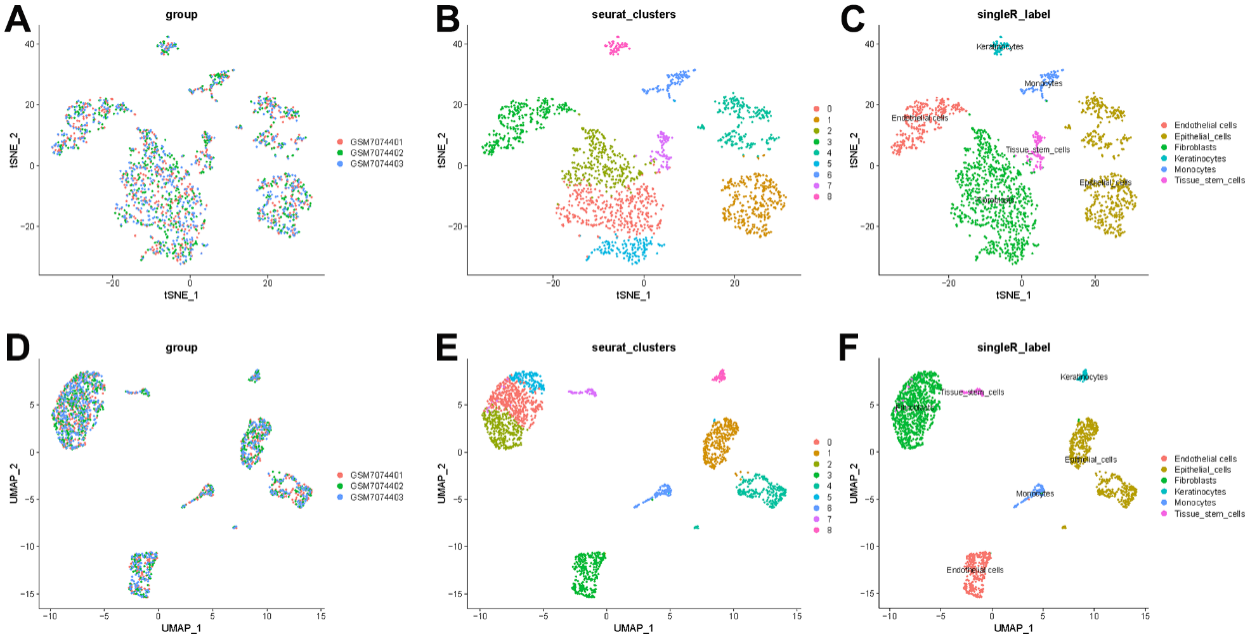
**t-SNE and UMAP clustering of normal breast samples.** (**A**) Inter-tumor heterogeneity of triple-negative breast cancer by t-SNE method. (**B**, **C**) Clusters and cell type annotations at the single-cell level in TNBC samples by t-SNE method. (**D**) Inter-tumor heterogeneity of triple-negative breast cancer by UMAP method. (**E**, **F**) Clusters and cell type annotations at the single-cell level in TNBC samples by UMAP method.

To better specify the types of these cells, we used the t-SNE and UMAP method to classify them into 14 clusters in TNBC, as shown in Figure 3A. The 14 clusters were labeled into eight cell types based on known marker expression, including epithelial cells, T cells, tumor-associated fibroblasts, macrophages, tumor stem cells, smooth muscle cells, tissue stem cells, and endothelial cells. The gene markers for each cell cluster are shown in the scatter plot in Figure 3B. There was heterogeneity in the distribution of cell types per tumor sample, as shown in Figure 3C. Whereas in normal breast, these cells were clustered by 9 clusters in t-SNE and UMAP, as shown in Figure 4. The 9 clusters were labeled into 6 types, including endothelial cells, epithelial cells, fibroblasts, keratinocytes, monocytes and tissue stem cells. Interestingly, we found that the cells labeled CAFs were divided into three subpopulations, whereas in normal breast tissue there was only one cluster of fibroblasts. We suggested that this may be due to heterogeneity within CAFs as well.

### Heterogeneity of CAFs and functional analyses

**Figure 5:**
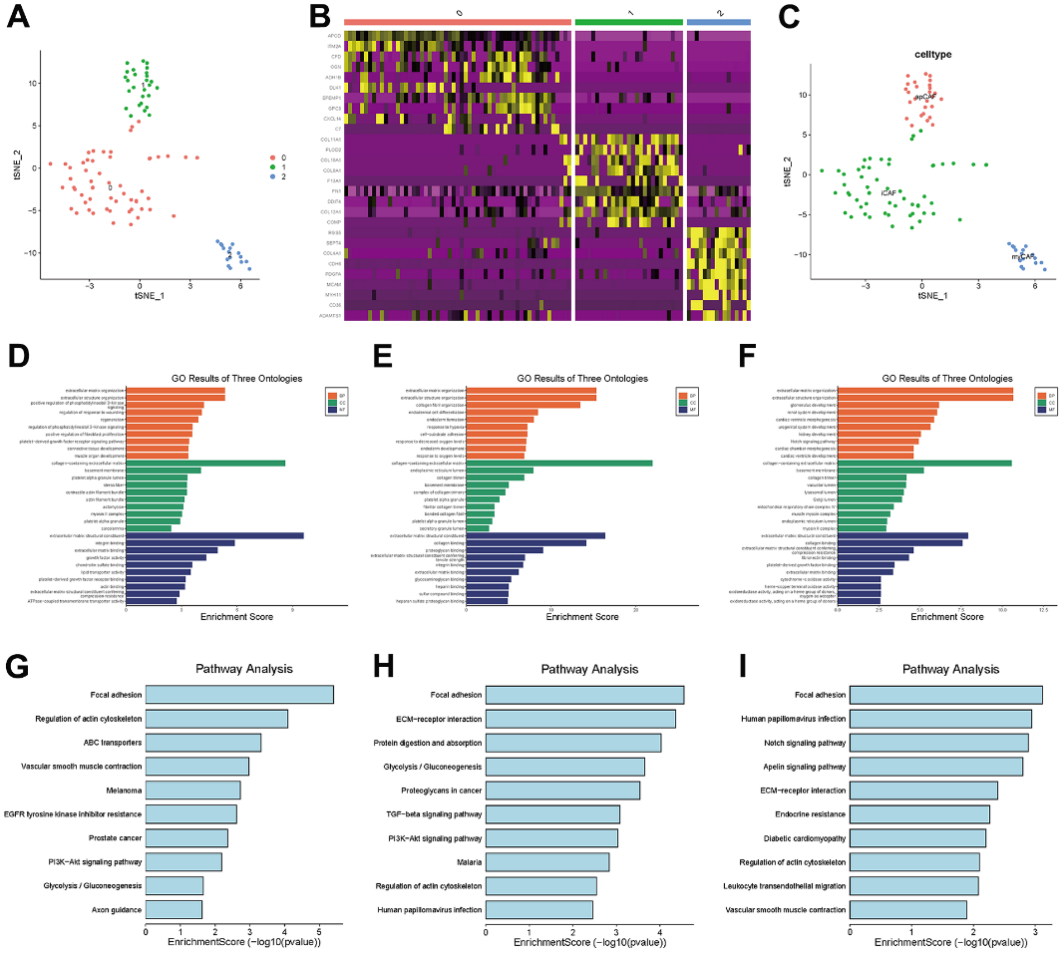
**CAF subgroup analysis.** (**A**) Scatter plot of the distribution of clusters in CAFs. (**B**) Heatmap of the distribution of gene markers in clusters. (**C**) Type annotation of CAF subgroups. (**D**) Results of GO analyses of prCAF subgroups. (**E**) Results of GO analyses of myCAF subgroups. (**F**) Results of GO analyses of emCAFs. (**G**) Results of KEGG pathway analyses of prCAF subgroups. (**H**) Results of KEGG pathway analyses of myCAF subgroups. (**I**) Results of KEGG pathway analyses of prCAF subgroups.

In TNBC, clusters 7, 11 and 13 were labeled CAFs, and the marker genes for these clusters were DCN, FAP and RGS5, respectively, which are widely reported markers of CAFs. We performed a separate t-SNE analysis of CAFs and found that they were clearly divided into three subgroups, as shown in Figure 5A. The marker genes for each subgroup can be seen in the heatmap in Figure 5B.

To identify the function of each subgroup, we performed GO and KEGG analyses to identify their functional differences, as shown in Figure 5D–5I. The cluster 0 subgroup is functionally characterized by promoting proliferation and multi drug resistance, activating PI3K signalling and ABC transporters, the cluster 1 subgroup is functionally characterized by constituting the extracellular matrix and collagen production, and the cluster 2 subgroup is functionally characterized by energy metabolism, in which Notch signaling pathway and Apelin pathway are activated. In the present research, we labeled cluster 0 as prCAFs, cluster 1 as myCAFs, and cluster 2 as emCAFs, as shown in Figure 5C.

### Molecular subgroup of TNBC based on CAF characteristic genes

**Figure 6:**
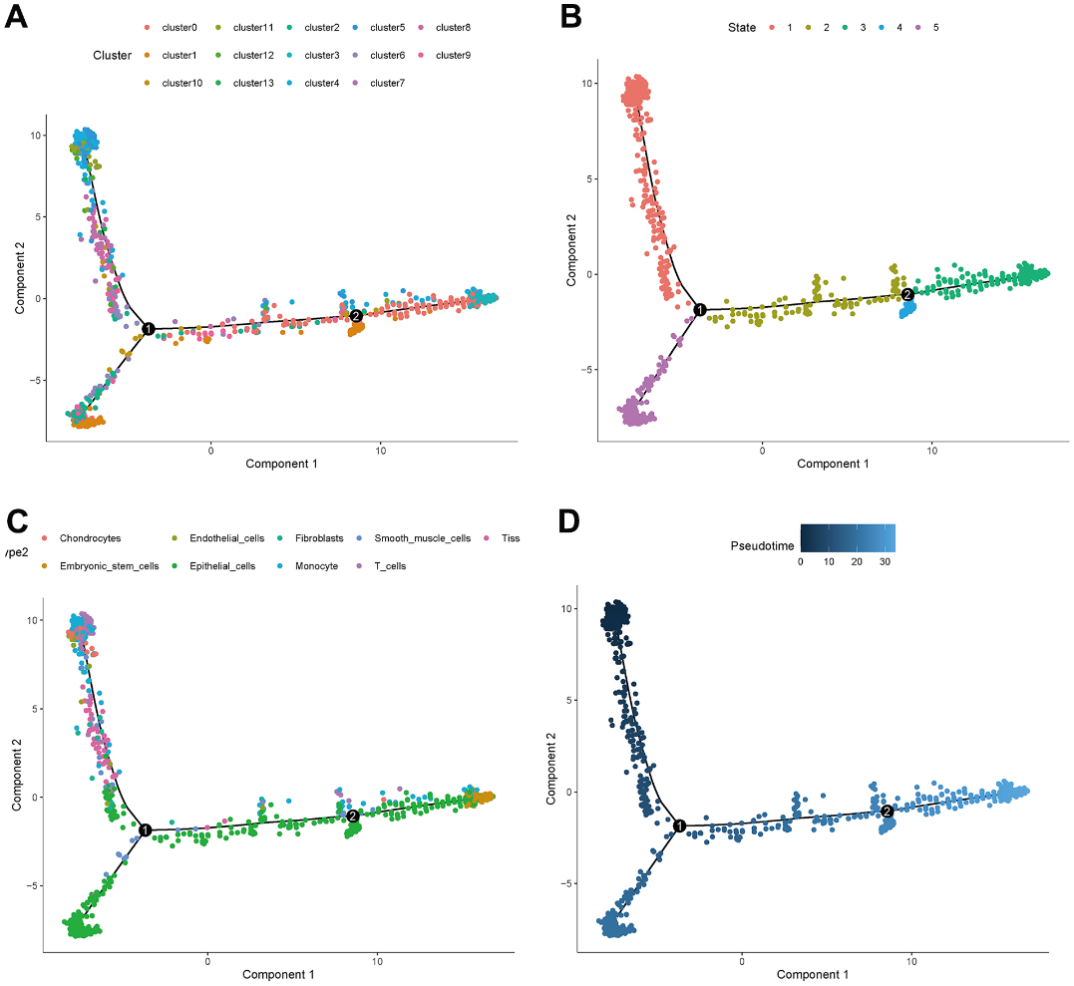
**Trajectory analyses of a single cell.** (**A**) Trajectory diagram of cell clusters. (**B**) Trajectory diagram of cell status. (**C**) Trajectory diagram of cell types. (**D**) Trajectory diagram of cell differentiation time.

The distribution of clusters in the trajectory diagram is shown in Figure 6A. The differentiation trajectory diagram showed that there are five main states of these cells (Figure 6B), and it can be seen that epithelial cells are distributed in all five states, and CAFs are mainly distributed in the first state, with the largest number of cell types in this state, as shown in Figure 6C. The pseudotime analysis diagram showed that CAFs are mainly distributed at the starting point from the pseudotime line, as shown in Figure 6D.

**Figure 7:**
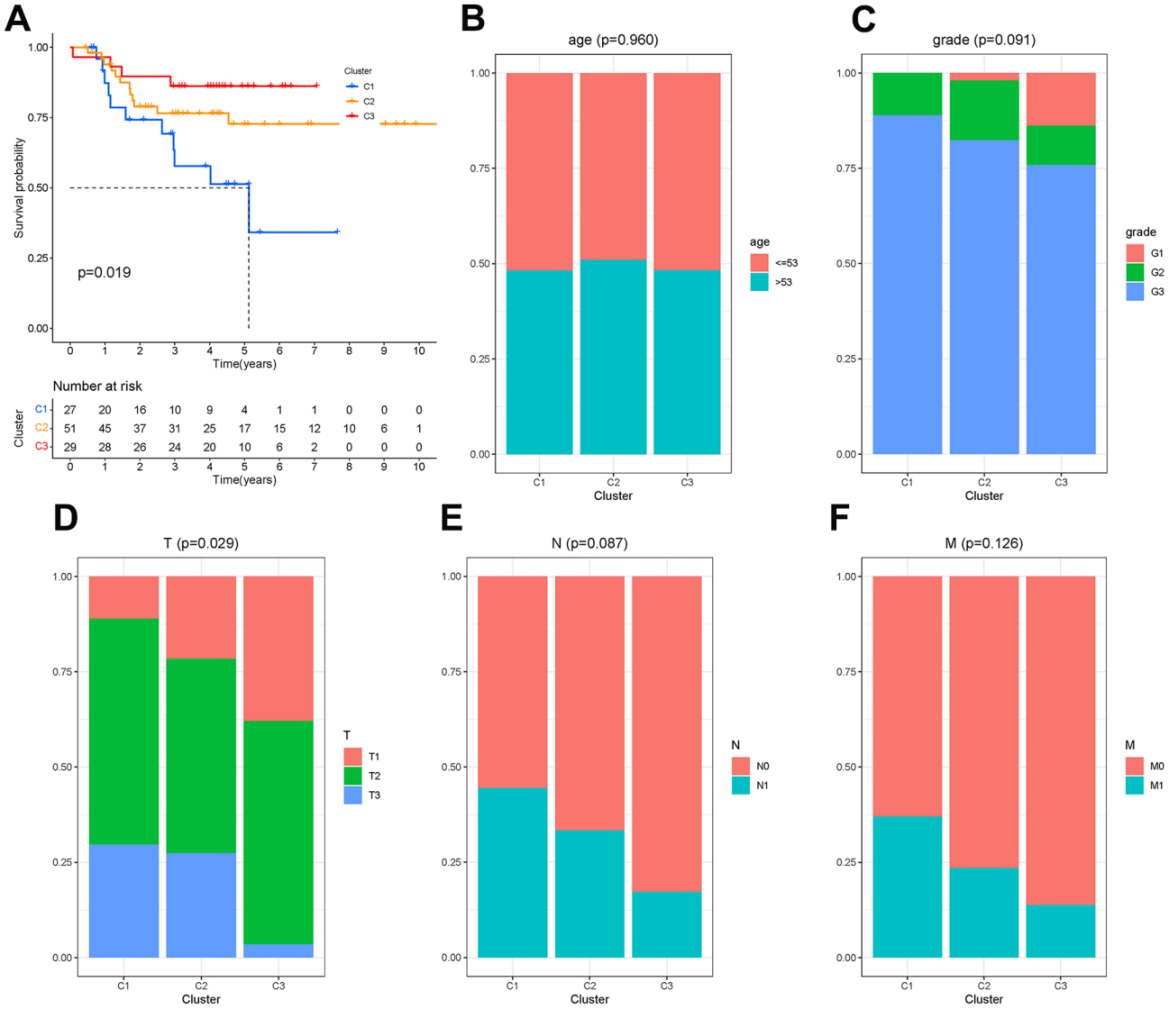
**Molecular clusters of TNBC.** (**A**) Kaplan-Meier analysis among molecular clusters. (**B**–**F**) Distribution of clinical characteristics in clusters.

There were 450 characteristic genes in the first state, and these genes were further analyzed. The 107 patients from GSM19615 and GSM21653 were divided into 3 clusters based on the expression of these genes. The clinical characteristics of patients were shown as Table 1. The Kaplan-Meier analysis (Figure 7A) showed that the survival probability of patients in cluster C3 was the longest and that of patients in cluster C1 was the shortest (P=0.019). Age, grade, lymph node metastasis (N) and distant metastasis (M) did not differ among the three clusters (P>0.05), while the size of the tumor (T) differed from each cluster (P=0.029), as shown in Figure 7B–7F.

**Table 1:** TNBC patient information (bulk RNA-seq).

**Characteristics**	**TCGA cohort N = 143**	**GEO cohort N = 107**
Age		
<=53	63(44.06%)	54(50.47%)
>53	80(55.94%)	53(49.53%)
Gender		
Female	143(100.00%)	107(100%)
Grade		
G1	0	5(4.67%)
G2	0	14(13.08)
G3	0	88(82.24%)
Unknown	143(100%)	0
Stage		
I	23(16.08%)	0
II	96(67.13%)	0
III	23(16.08%)	0
IV	1(0.01%)	0
Unknown	0	107(100%)
Survival status		
Alive	124(86.71%)	80(14.62%)
Dead	19(13.29%)	27(85.38%)
The median follow-up time (year)	2.12	4.02

### Tumor immune microenvironment (TIME) differs between clusters

**Figure 8:**
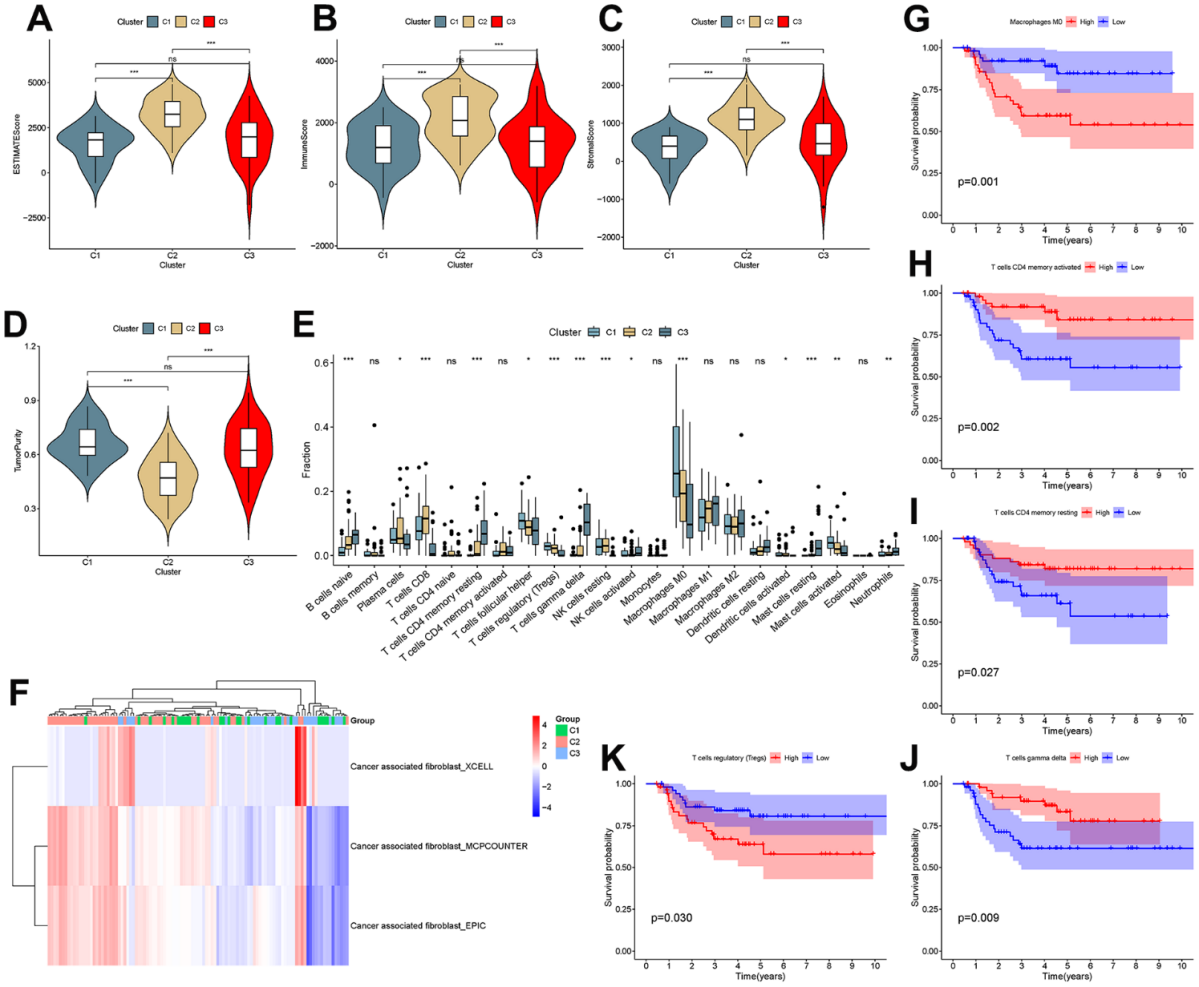
**Analysis of the tumor microenvironment among molecular subtypes.** (**A**–**D**) ESTIMATE score, immune score, stromal score and tumor purity among clusters. (**E**) Comparison of infiltration of 22 immune cells among clusters. (**F**–**J**) Immune cells associated with survival in triple-negative breast cancer patients.

To investigate the correlation between cluster and TIME, ESTIMATE and CIBERSORT analyses were performed. The results showed that there were differences in the ESTIMATE score, immune score, stromal score and tumor purity between C1 and C2 and between C2 and C3 (P<0.001), as shown in Figure 8A–8D. This suggested that there are differences in the TIME and stromal microenvironment among clusters. Among the three clusters, C2 had the least tumor cell infiltration and the most immune cells and stromal components. There were differences between clusters in multiple immune cells, including T cells, as shown in Figure 8E. Calculation of CAF scores in the tumor microenvironment using the Xcell, MCPcounter and EPIC algorithms is shown in Figure 7F. Kaplan-Meier analysis showed that CD4 memory activated T cells, CD4 memory resting T cells and gamma delta T cells were positively related to overall survival, while regulatory T cells and M0 macrophages were negatively related to overall survival (Figure 8G–8K).

**Figure 9:**
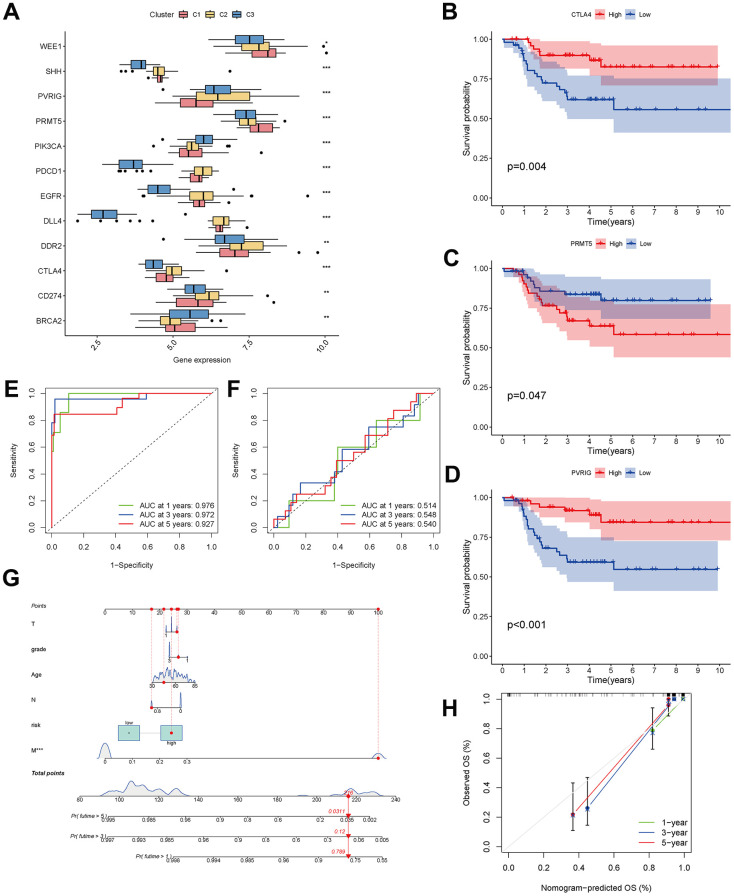
**Analysis of CAF-related genes between molecular subtypes.** (**A**) The expression of immune checkpoint-related genes among clusters. (**B**–**D**) The expression of checkpoint-related genes associated with survival in triple-negative breast cancer patients. (**E**, **F**) ROC curves for the training and testing sets. (**G**) Nomogram predicting patient survival prognosis. (**H**) Calibration curve of the nomogram.

A checkpoint distribution among the three clusters was performed to predict the response to immunotherapy. There were differences among clusters in the distribution of genes encoding the immune checkpoints WEE1, SHH, PVRIG, PRMT5, PIK3CA, PDCD1, EGFR, DLL4, DDR2, CTLA4, CD274 and BRCA (Figure 9A). In Kaplan-Meier analyses, high expression of CTLA4 and PVRIG and low expression of PRMT5 were related to longer overall survival (Figure 9B–9D).

### Development of a prognostic predictive model and a nomogram

**Figure 10:**
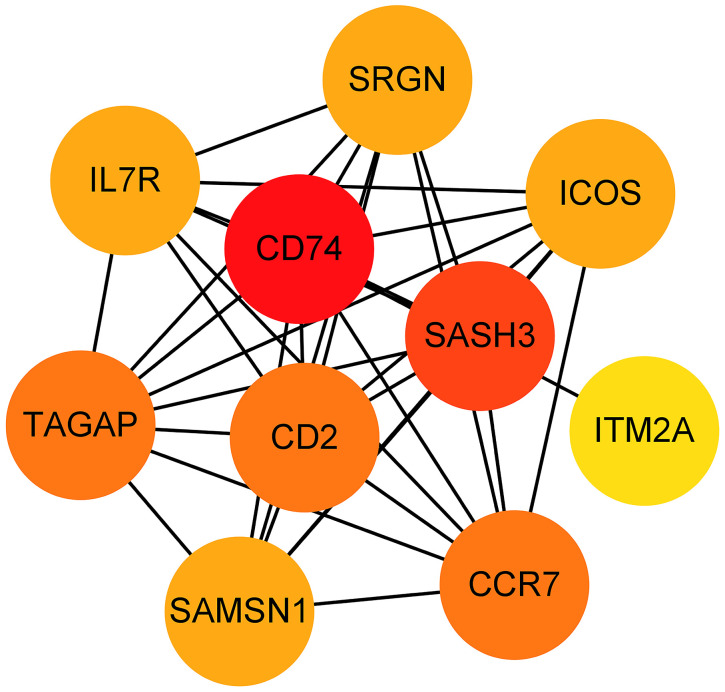
**Hub genes obtained from the prognostic predictive model.** Note: The dots represent proteins, the lines represent the interaction between proteins, and the color of the dots represents the degree (red: high; yellow: low).

The 33 genes associated with prognosis were screened using univariate regression analysis and then compressed to 16 genes using LASSO regression, shown as Table 2. The AUC values of the ROC curves in the training and test sets validate that the model is stable. The 1-year AUC of the training set was 0.976, the 3-year AUC was 0.972, and the 5-year AUC was 0.927 (Figure 9E). The 1-year AUC of the testing set was 0.514, the 2-year AUC was 0.548, and the 3-year AUC was 0.540 (Figure 9F). Univariate and multivariate Cox regressions were implemented for clinical characteristics and risk scores, respectively, and the results showed that the risk score was related to overall survival (univariate: HR= 1.007, 95% CI (1.004−1.009), P<0.001; multivariate: HR= 1.005, 95% CI (1.002−1.007), P<0.001). To visualize the model, a nomogram was applied (Figure 9G). Calibration curves showed that the nomogram predicted 1-year survival more accurately and may underestimate 3- and 5-year survival (Figure 9H). The hub genes were CD74, SASH3, CD2, TAGAP and CCR7 by degree of nodes (Figure 10).

**Table 2:** CAF-related genes to construct a prognostic model.

**Gene name**	**Coefficient**	**HR**	**HR.95 L**	**HR.95H**	**pvalue**
SRGN	1.55	4.72	1.47	15.12	0.01
ITM2A	1.17	3.21	1.02	10.09	0.05
CD74	-0.59	0.55	0.27	1.15	0.11
SAMSN1	-2.58	0.08	0.01	0.52	0.01
CD2	0.91	2.49	0.69	8.91	0.16
IL7R	0.63	1.89	0.86	4.18	0.12
SASH3	1.75	5.76	1.31	25.44	0.02
TAGAP	1.94	6.96	1.42	34.10	0.02
CCR7	-2.22	0.11	0.02	0.53	0.01
ICOS	-3.42	0.03	0.01	0.18	<0.001
ALDOA	2.14	8.49	2.82	25.56	<0.001
MRFAP1	2.33	10.23	2.74	38.15	<0.001
LCLAT1	2.19	8.96	2.19	36.73	<0.001
PCNA	1.41	4.11	1.52	11.10	0.01
MAN2B1	-0.94	0.39	0.14	1.10	0.08
ZNF445	1.83	6.23	1.67	23.29	0.01

## DISCUSSION

Breast cancer is a highly heterogeneous disease and this heterogeneity leads to the development of drug resistance and progression [17], in order to reveal the source of this heterogeneity as well as to explore therapeutic targets, our study was implemented. In the presented research, the intra- and inter-tumor heterogeneity was identified, and three subpopulations of CAFs and five branches in the differentiation trajectory were recognized by the scRNA-seq data. The three subgroups are prCAFs, which promote proliferation and multi drug resistance; myCAFs, which are involved in constituting the extracellular matrix and collagen production; and emCAFs, which are functionally characterized by energy metabolism. The naming of these subpopulations was proposed only for the present study, and we looked for established subpopulation names that matched the results of the present study as closely as possible, and for the other subpopulations, naming them according to their functions. The differentiation trajectory showed five statuses of cells. We found that CAFs were enriched in branch 1 and appeared in the early stages of differentiation. We divided the patients into three clusters based on the differentially expressed genes in the branch containing CAFs. In Kaplan-Meier analysis, C3 patients had a higher survival probability, C2 had the second highest survival probability and C1 had the lowest survival probability. The clinical features and TIME were characterized by clusters. Tumor size and tumor microenvironment varied among clusters. There were differences in T cells and M0 macrophages among clusters. High infiltration of CD4+ memory activated T cells, CD4+ memory resting T cells and gamma delta T cells was related to higher survival probability, while low infiltration of M0 macrophages and regulatory T cells was related to higher survival probability. Immune check-point-related genes varied among clusters. In addition, a prognostic model and nomogram were developed and verified.

In the scRNA-seq data, we found that the sample consisted mainly of epithelial cells, and cancer cells were almost concentrated in the epithelial cells. This result is consistent with the fact that breast cancer originates from the epithelial cells of the breast. Other cells within the sample were cancer-associated fibroblasts, tissue stem cells, smooth muscle cells, embryonic stem cell-like cells, endothelial cells, macrophages and T cells. Cancer-associated fibroblasts were clearly divided into three clusters, and their marker genes were DCN, FAP and RGS5. DCN is considered a marker for one of these subgroups in CAFs of colorectal cancer and high-grade serous ovarian cancer samples [27, 28]. FAP is thought to be a CAF-specific expression protein [29]. RGS5 serves as the gene marker of myCAFs [30, 31]. Single-cell RNA sequencing in patients with intrahepatic cholangiocarcinoma divides CAFs into inflammatory and growth factor-rich and myCAF subpopulations, showing distinct ligand-receptor interactions. myCAFs express hyaluronate synthase 2, whereas type I collagen is not expressed and promotes intrahepatic cholangiocarcinoma [32]. The results were consistent with the results of our study. Six CAF subpopulations have been identified in 4T1 tumors, including 1) myofibroblast CAFs, enriched in α-SMA54re and several other contractile proteins; 2) CAFs with elevated expression of “inflammatory” inflammatory cytokines; and 3) CAF subpopulations expressing major histocompatibility complex class II proteins, which are typically expressed in antigen-presenting cells [23]. The expression of activated CAF-specific genes significantly correlated with patient survival [33]. Four major subgroups of fibroblasts were identified in gastric cancer samples: myofibroblasts, pericytes, extracellular matrix CAFs and immunomodulatory CAFs [34]. The function of each subgroup is different. High iCAF levels may indicate a high degree of malignancy, while high myCAF levels may indicate a poor response to treatment [30]. ICAFs appear to promote tumors better than myCAFs by producing chemokines and cytokines [35]. myCAFs may deposit large amounts of ECM to impede drug delivery [36]. In breast cancer, scRNA-seq in mouse models detected four distinct CAF phenotypes [37], termed vascular CAFs, matrix CAFs, cycling CAFs and developmental CAFs [37]. A scRNA-seq study using a triple-negative breast cancer mouse model further identified 2 CAF phenotypes, pCAF and sCAF, showed that CAFs functions change with tumor progression [38]. Our results for CAFs subgroups differ from the previous studies in perspective of concern, which focused more on the relationship between CAFs and immune cells, whereas the present study identified the pathways and mechanisms by which CAFs acts. Lena Cords also provided 8 CAF classification: vascular CAFs, matrix CAFs, immune CAFs, tumor-like CAFs, interferon-response CAFs, antigen-presenting CAFs, dividing CAFs, and reticular-like CAFs [39]. For triple-negative breast cancer, 52% of patients have low peripheral lymphocyte infiltration and poor PD-1/PD-L1 treatment [40], which is supported by the single-cell clustering results of the presented research, so targeting the relevant pathways and mechanisms may create a breakthrough.

The CAFs marker FAP mentioned in the presented study has been the subject of several preclinical studies and is considered a promising target. Treatment of xenograft models of lung, pancreatic and head and neck cancers with a novel antibody-metanicinol conjugate, FAP5-DM1, resulted in long-term inhibition of tumor growth and complete regression with no signs of intolerance [41]. Depletion of FAP-positive stromal cells with the FAP-targeted immunotoxin αFAP-PE38 reduced the recruitment of tumor-infiltrating immune cells in the tumor microenvironment and inhibited tumor growth [42]. There are also novel FAP-targeted immunotherapies, such as DNA vaccination [43], chimeric antigen receptor T cells [44, 45] or lysing adenovirus [46, 47]. The FAP-specific antibody sibrotuzumab was considered clinically safe and effective in phase I trials in advanced cancer [48, 49]. However, no beneficial effects were shown in phase II trials in metastatic colorectal cancer [50].

Interestingly, we found a cluster of embryonic stem cell-like cells in these triple-negative breast cancer samples. Poorly differentiated basal-like breast cancer with similarities in gene expression to embryonic and induced pluripotent stem cells [51]. The extensive proliferation, migration and invasion required for mammogenesis do not occur in the resting adult breast, but they do resemble the processes that mediate breast cancer progression. Forced coexpression of luminal keratins 8 and 18 with vimentin in human breast cancer cells *in vitro* increases motility, invasiveness, and proliferation [52]. Similarly, basal-like breast cancers frequently exhibit an undifferentiated phenotype and coexpress keratin and wave proteins of the myoepithelium and tubular epithelium [53]. The epithelial-to-mesenchymal transition commonly observed during invasive tumorigenesis may represent a return to an embryonic-like state and may also promote a stem cell-like state in breast cells [54, 55]. Based on the above studies, we speculated that this fraction of cells may be embryonic stem cell-like mammary epithelial cells generated by the EMT process due to tumor tissue generation.

We identified 5 hub genes, including CD74, SASH3, CD2, TAGAP and CCR7. CD74 is a type II transmembrane protein that is primarily expressed on antigen-presenting cells and functions intracellularly as a major histocompatibility complex class II chaperone [56, 57]. Its expression is highly correlated with tumor progression, especially in chronic lymphocytic leukemia [58]. SASH3 has also been reported by other bioinformatic analyses as a tumor microenvironment-related gene with prognostic value for breast cancer [59]. SASH3 is important for T-cell proliferation, activation and cell survival, and deficiency or mutation of SASH3 may lead to a decrease in CD4 T-cell lymphocytes, a decrease in T-cell proliferation, a decrease in cell cycle progression and an increase in T-cell apoptosis [60]. Our findings suggested that T-cell infiltration differed among clusters. CD2 promotes adhesion and signaling and counteracts human T-cell depletion. Low CD2 expression is shown in patients with endometrial or ovarian cancer. CD2 downregulation may attenuate antitumor T-cell responses and have implications for checkpoint immunotherapy [61]. In weighted gene co-expression network analysis, TAGAP was considered a key marker associated with prognosis in bladder cancer [62], endometrial cancer [63], and prostate cancer [64]. In tumors, CCL chemokine expression is present in cancer-associated fibroblasts [65]. The CCR7 chemokine axis plays an important role in controlling the migration of tumor cells to the lymphatic system and metastasis and therefore contributes to cancer expansion [66]. In esophageal squamous carcinoma, activation of CCR7 increases VEGF-A expression in cancer cells by increasing angiogenesis through activation of NF-κB [67]. It also causes EMT and migration of cancer cells [68, 69].

In this study, three CAF subgroups and marker genes were identified, their functions were annotated, and a prognostic model based on CAF-related genes was constructed. However, some limitations in our study should be acknowledged. Clinical information corresponding to single-cell data was not available for this study, resulting in failure to analyze the relationship between CAF percentage and survival in individual samples. The functions of the CAF subgroup proposed in this study need to be verified by biological tests. In the future, we will perform flow cytometry to classify CAFs and investigate their effects on the proliferation, migration and apoptosis of TNBC cells.

## CONCLUSIONS

In summary, we identified three subgroups: prCAFs (marked by DCN), myCAFs (marked by FAP) and emCAFs (marked by RGS5). Their functions are promoting angiogenesis, constructing matrix composition and collagen production, having myofibroblast characteristics and inducing endocrine resistance. CAFs are a source of inter- and intra-tumor heterogeneity in TNBC tumors. A prognostic model based on CAF-related genes was constructed and verified by a testing set. Five hub genes, CD74, SASH3, CD2, TAGAP and CCR7, served as significant marker genes with prognostic value.

## Supplementary Material

Supplementary Figures
