# Supplementary material for: Integrated analysis of single-cell RNA-seq and bulk RNA-seq unravels the heterogeneity of cancer-associated fibroblasts in TNBC
Source: Aging (Albany NY). 2023 Nov 13;15(21):12674–97. doi: 10.18632/aging.205205 (PMC10683606; doi:10.18632/aging.205205)
Supplement: Supplementary Figures [file aging-15-205205-s001.pdf]

SUPPLEMENTARY FIGURES

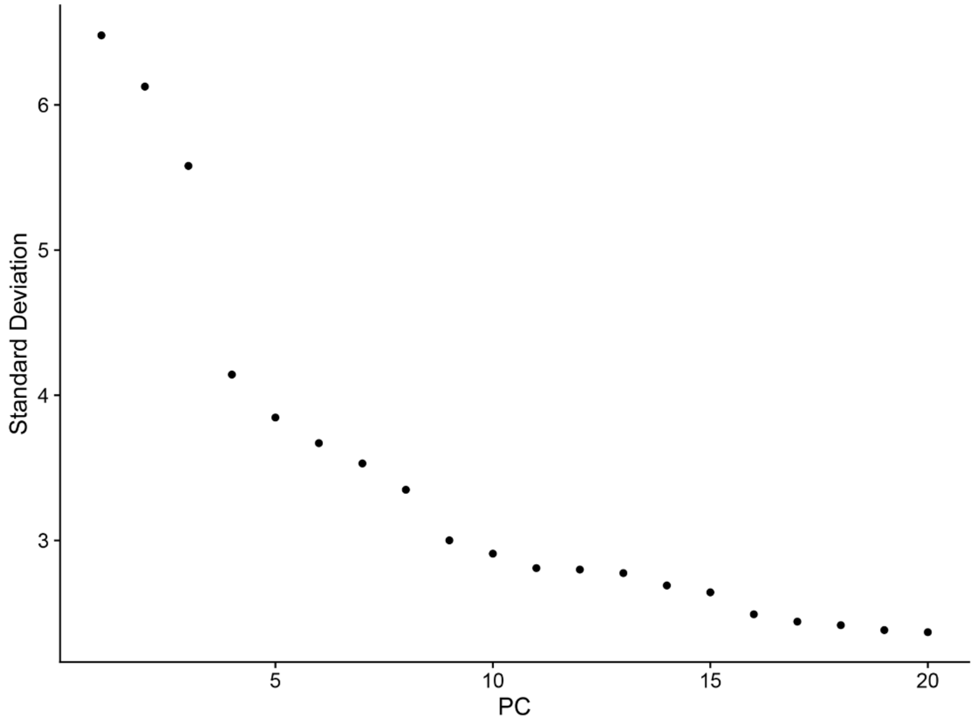

Supplementary Figure 1. Elbow plot of PCA in normal breast tissue data.

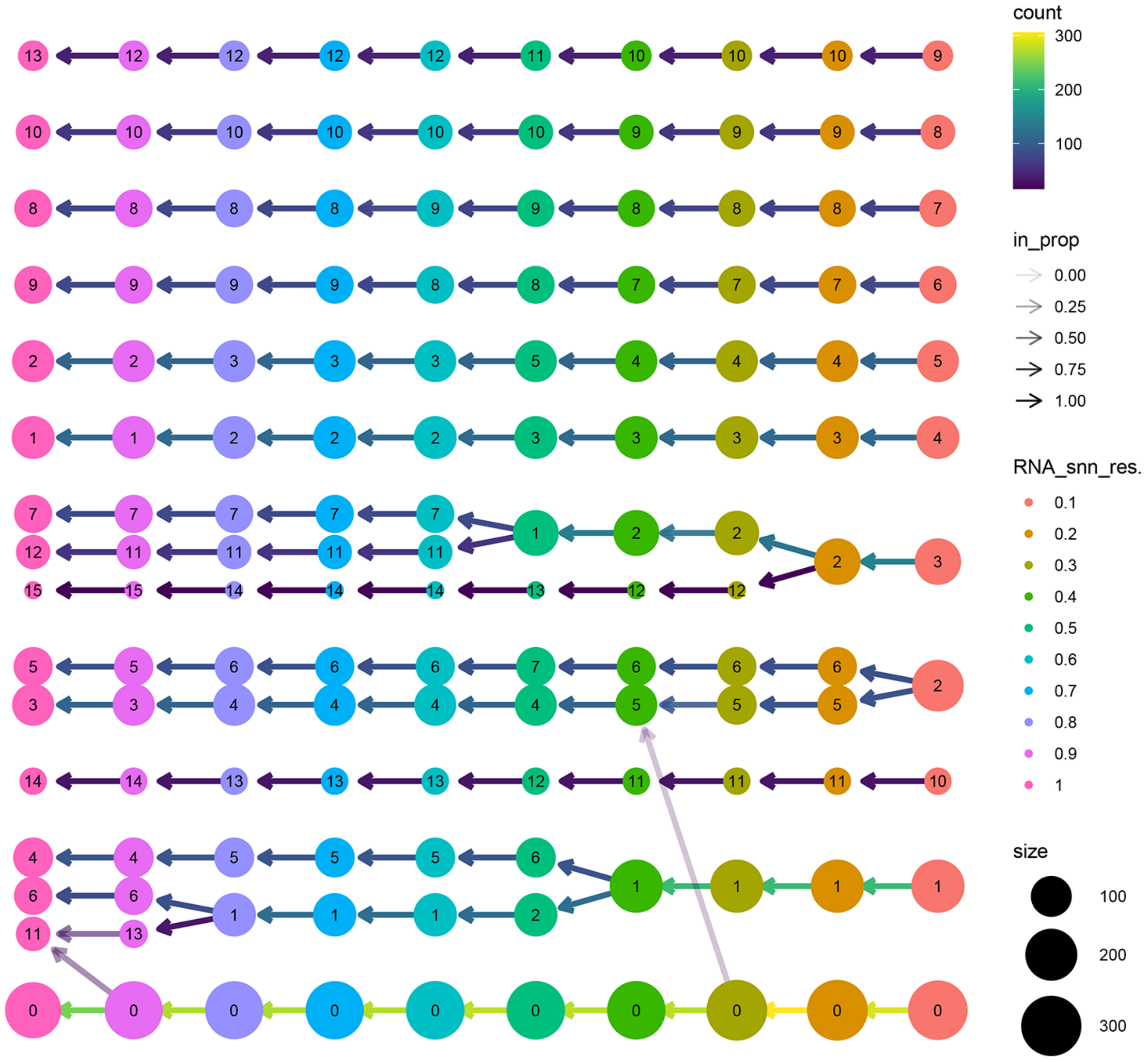

Supplementary Figure 2. Plot of identification of resolution in normal breast tissue data.

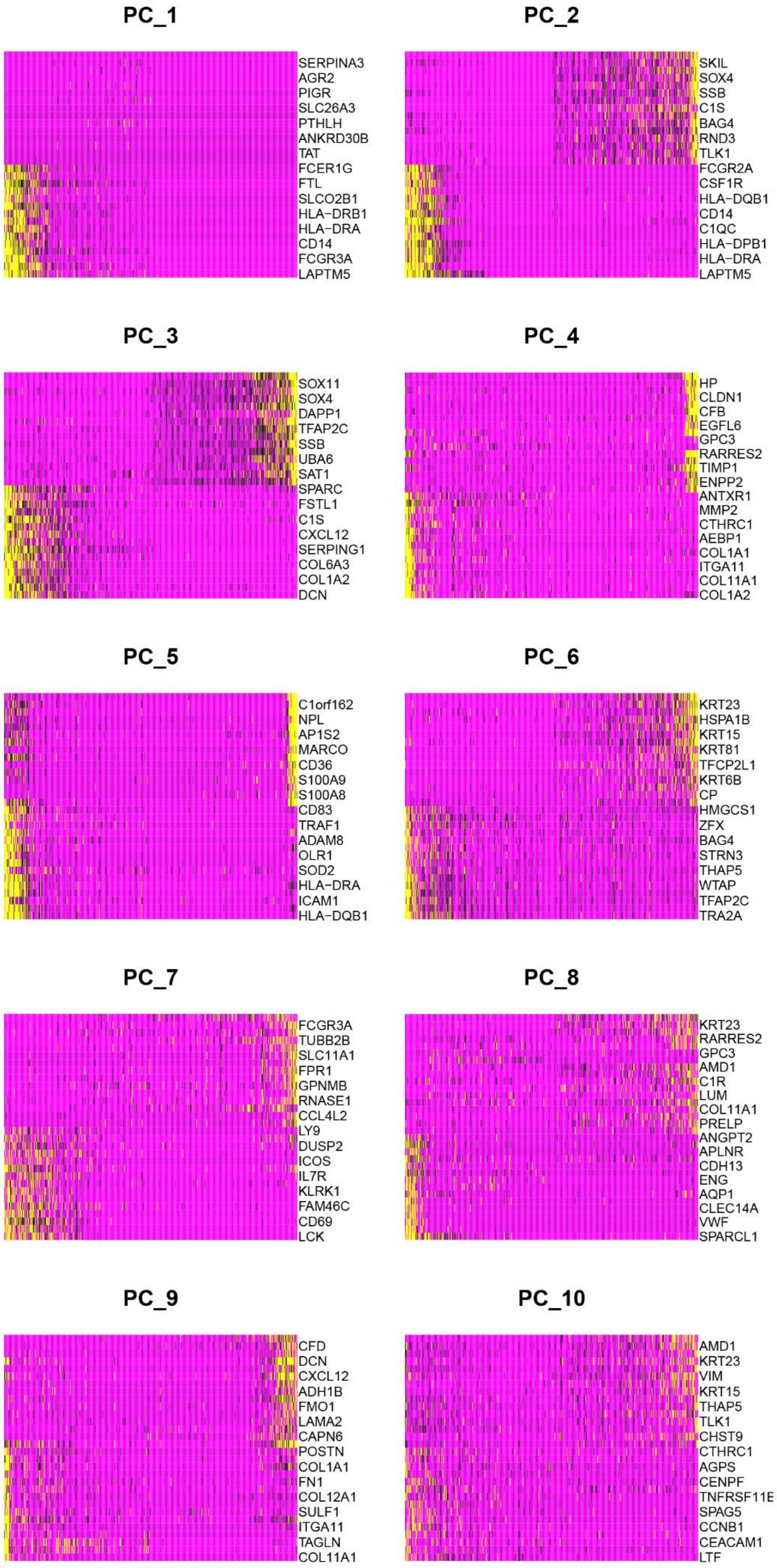
PC_1
SERPINA3
AGR2
PIGR
SLC26A3
PTHLH
ANKRD30B
TAT
FCER1G
FTL
SLCO2B1
HLA−DRB1
HLA−DRA
CD14
FCGR3A
LAPTM5
PC_2
SKIL
SOX4
SSB
C1S
BAG4
RND3
TLK1
FCGR2A
CSF1R
HLA−DQB1
CD14
C1QC
HLA−DPB1
HLA−DRA
LAPTM5
PC_3
SOX11
SOX4
DAPP1
TFAP2C
SSB
UBA6
SAT1
SPARC
FSTL1
C1S
CXCL12
SERPING1
COL6A3
COL1A2
DCN
PC_4
HP
CLDN1
CFB
EGFL6
GPC3
RARRES2
TIMP1
ENPP2
ANTXR1
MMP2
CTHRC1
AEBP1
COL1A1
ITGA11
COL11A1
COL1A2
PC_5
C1orf162
NPL
AP1S2
MARCO
CD36
S100A9
S100A8
CD83
TRAF1
ADAM8
OLR1
SOD2
HLA−DRA
ICAM1
HLA−DQB1
PC_6
KRT23
HSPA1B
KRT15
KRT81
TFCP2L1
KRT6B
CP
HMGCS1
ZFX
BAG4
STRN3
THAP5
WTAP
TFAP2C
TRA2A
PC_7
FCGR3A
TUBB2B
SLC11A1
FPR1
GPNMB
RNASE1
CCL4L2
LY9
DUSP2
ICOS
IL7R
KLRK1
FAM46C
CD69
LCK
PC_8
KRT23
RARRES2
GPC3
AMD1
C1R
LUM
COL11A1
PRELP
ANGPT2
APLNR
CDH13
ENG
AQP1
CLEC14A
VWF
SPARCL1
PC_9
CFD
DCN
CXCL12
ADH1B
FMO1
LAMA2
CAPN6
POSTN
COL1A1
FN1
COL12A1
SULF1
ITGA11
TAGLN
COL11A1
PC_10
AMD1
KRT23
VIM
KRT15
THAP5
TLK1
CHST9
CTHRC1
AGPS
CENPF
TNFRSF11E
SPAG5
CCNB1
CEACAM1
LTF

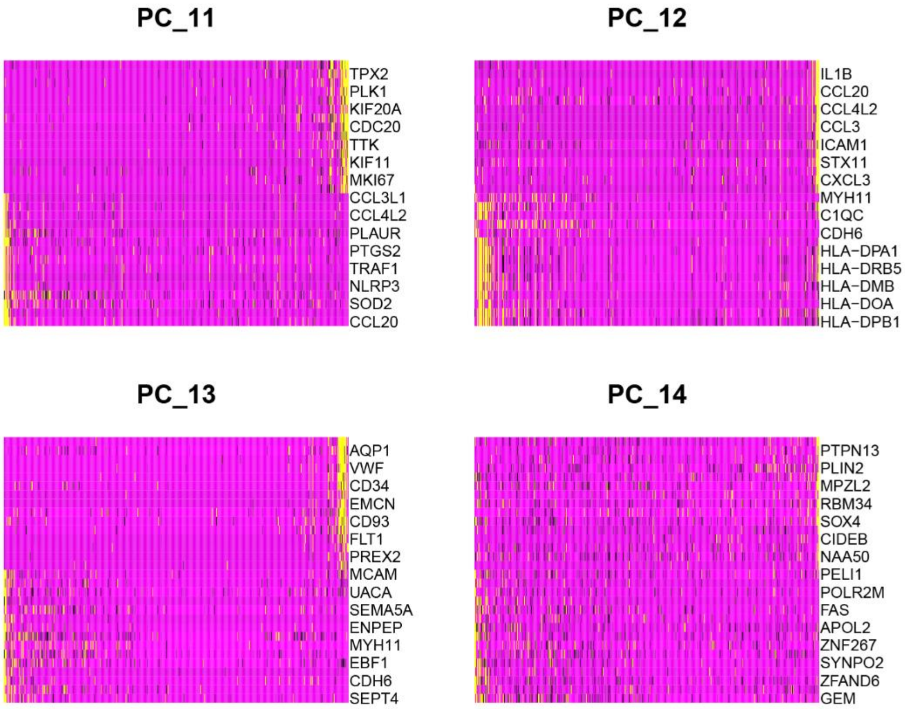

Supplementary Figure 3. Heatmap plot of PCA in TNBC data.

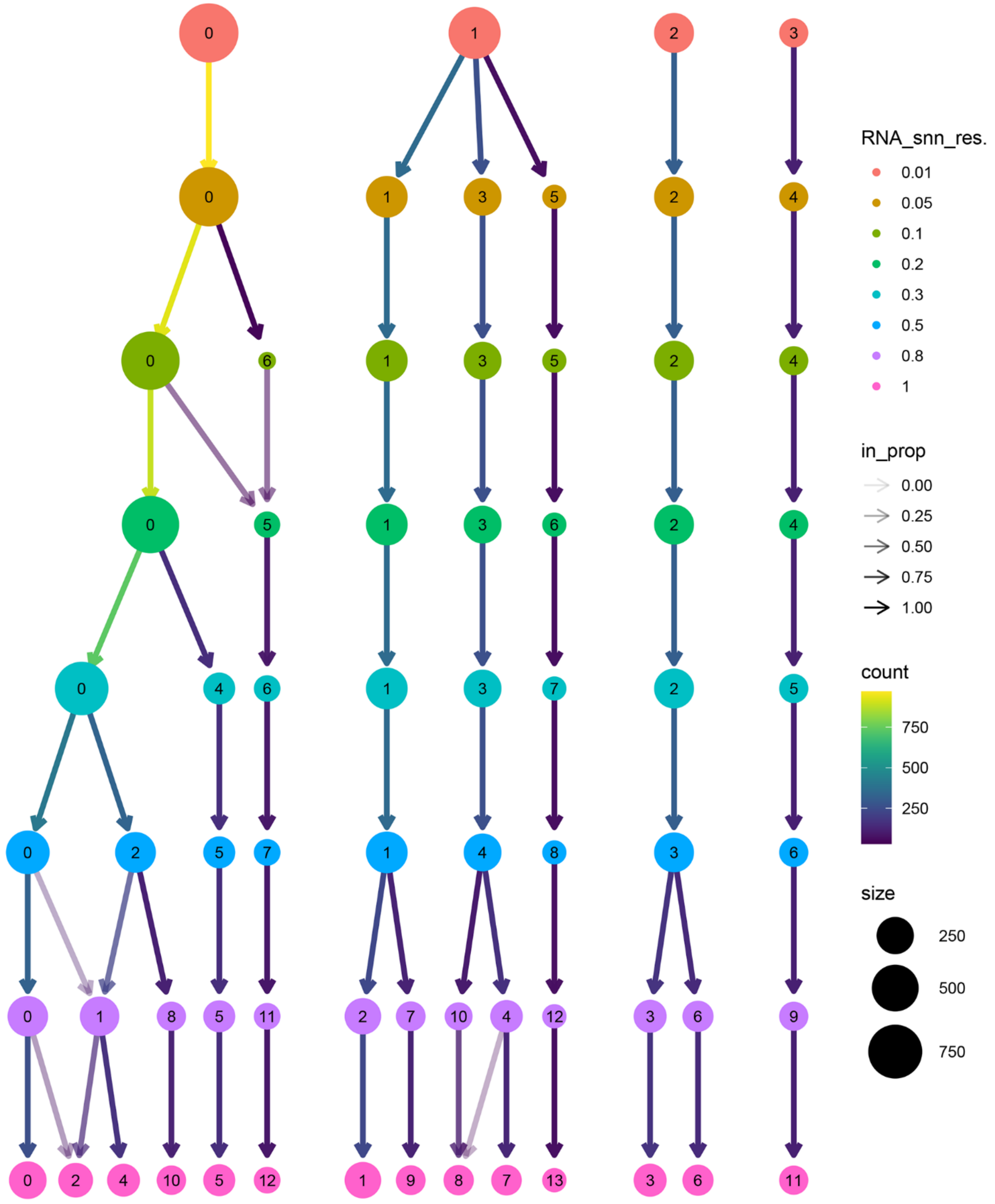

Supplementary Figure 4. Plot of identification of resolution in TNBC data.
